# Recruiting ‘hard to reach’ parents for health promotion research: experiences from a qualitative study

**DOI:** 10.1186/s13104-021-05653-1

**Published:** 2021-07-21

**Authors:** Louise Tully, Eleni Spyreli, Virginia Allen-Walker, Karen Matvienko-Sikar, Sheena McHugh, Jayne Woodside, Michelle C. McKinley, Patricia M. Kearney, Moira Dean, Catherine Hayes, Caroline Heary, Colette Kelly

**Affiliations:** 1 Health Promotion Research Centre, National University of Ireland, Galway, Ireland; 2 Centre for Public Health, School of Medicine, Dentistry and Biomedical Sciences, Queens University Belfast, Belfast, UK; 3 School of Biological Sciences, Institute for Global Food Security, Queens University Belfast, Belfast, UK; 4 School of Public Health, University College Cork, Cork, Ireland; 5 School of Medicine, Trinity College Dublin, Dublin, Ireland; 6 School of Psychology, National University of Ireland, Galway, Ireland

**Keywords:** Social Determinants of Health, Research recruitment, Health promotion research, Parenting, Marginalized groups, Hidden populations, Seldom heard voices, Qualitative research

## Abstract

**Objective:**

Marginalised populations are less likely to take part in health research, and are sometimes considered ‘easy to ignore’. We aimed to describe our approach and results of recruiting parents who experience disadvantage, for focus groups exploring infant feeding on the island of Ireland. Upon receiving ethical approval, we implemented recruitment strategies that included building rapport with community organisations through existing networks, targeting specific organisations with information about our aims, and utilising social media groups for parents.

**Results:**

We approached 74 organisations of which 17 helped with recruitment. We recruited 86 parents/carers (one male) for 19 focus groups (15 urban/4 rural). Seventy two percent met at the eligibility criteria. Most participants were recruited through organisations (91%), and the remainder on social media (9%). Recruitment barriers included multiple steps, research fatigue, or uncertainty around expectations. Factors such as building rapport, simplifying the recruitment process and being flexible with procedures were facilitators. Despite comprehensive, multi-pronged approaches, the most marginalised parents may not have been reached. Further alternative recruitment strategies are required for recruiting fathers, rural populations, or those without the capacity or opportunity to engage with local services.

**Supplementary Information:**

The online version contains supplementary material available at 10.1186/s13104-021-05653-1.

## Introduction

Recruiting a diverse population for qualitative research, who have both experienced the phenomenon of interest and meet specific characteristics, can be challenging, particularly groups that are socially and/or economically disadvantaged, vulnerable or ‘hidden’ [1, 2].

We define ‘disadvantaged’ as those experiencing socio-economic disadvantage (i.e. at risk of inequality related to employment, education, income, access to healthcare/resources) and those at risk of social disadvantage (e.g. young parents, lone parents, migrants, those from ethnic minorities, people with disabilities). Those who are vulnerable due to inequality may experience an array of unique circumstances that results in their exclusion from health promotion research, enhancing the issue of ‘seldom heard voices’ [3] and potentially further exacerbates health inequalities [4, 5]. The term ‘easy to ignore’ rather than ‘hard to reach’ has been used to describe such groups, given these complexities [6], and a need for transparent accounts of researchers’ experiences of engaging with such groups for research has been identified [7].

We describe our experience of recruiting parents for qualitative health promotion research, and considerations for future recruitment for research aimed at similar populations. We recruited disadvantaged parents, with an infant aged 3–14 months, for focus groups to explore the barriers and facilitators for following infant feeding recommendations, the results of which are published elsewhere [8, 9].

## Main text

### Methods

We considered the evidence that social support during parenting predicts successful child outcomes [10–12] alongside the social determinants of health [13], in outlining our eligibility criteria (full criteria in Additional file 1: Table S1).

**Fig. 1:**
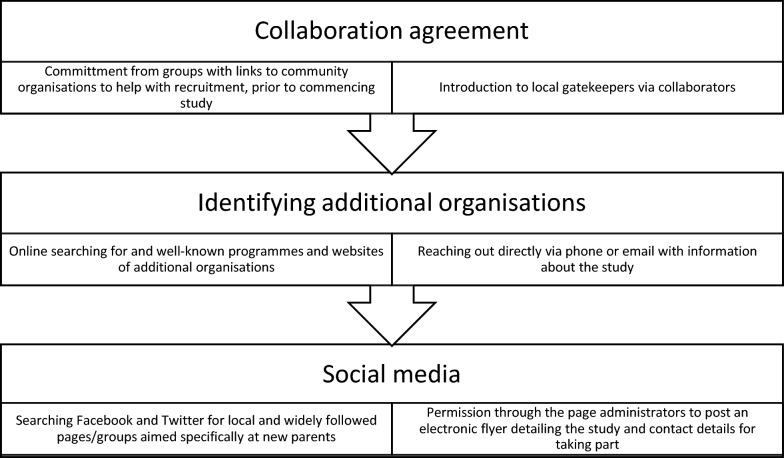
Three main recruitment strategies

The research team planned three recruitment strategies (Fig. 1). The first involved recruitment through local community organisations utilising a collaboration agreement that included researchers at a range of academic institutions, with links to the local community, and detailed a commitment to supporting the project. This facilitated additional key recruitment contacts such as local support workers involved directly with groups in deprived areas. Our strategy was to reach out to key gatekeepers within such organisations and, with their permission, provide the study materials to potential participants. We also searched online for key community organisations in the country that engaged specifically with disadvantaged parents, with the aim of employing purposive and snowball sampling. When organisations were identified, we contacted them via email or phone, and provided information about the study. Many of these organisations focused on parenting skills, baby care or support and personal development for single parent families.

For online recruitment of study participants, we shared study information within Facebook support groups for parents and families. The social media strategy included posting an e-flyer with details of the study and the eligibility criteria, asking parents to get in contact via phone, text message or email if they were interested in taking part, on multiple local and national parenting Facebook group pages and Twitter. They were subsequently invited to a focus group if interested.

To overcome documented barriers for attendance at focus groups related to location, timing, childcare responsibilities, format and structure, and cost implications [14], we informed gatekeepers that the focus group would be held at a community venue that is local and familiar to the parents. We also made clear that they would be welcome to bring along their baby, it would be an informal environment with refreshments provided, and where needed, participants would be reimbursed for travel costs. We described the data collection as a ‘chat’ to convey a sense of informality. We also gave a small gratuity voucher (€20/£20 multi-store gift cards) after focus groups to thank parents for their time and a booklet containing up-to-date recommendations on infant feeding relevant to the local jurisdiction. We gathered demographic and participant characteristic data using questionnaire items from a variety of sources including past censuses for each jurisdiction, and previous studies such as Growing up in Ireland [15]. The questionnaire used in ROI is available to view in supplementary material (Additional file 2), while the focus group topic guide is published elsewhere [16].

### Results

In the ROI, we recruited 46 parents/carers (45 female, one male) for participation in 11 focus groups, of which nine took place in urban and two in rural areas. These took place over seven months. A total of 37 mothers were recruited and took part in 8 NI focus groups, six of which took place in urban areas and two in rural. Focus groups spanned over a 4-month period. There were no fathers/male carers recruited to the NI sample. In both NI and the ROI, the first focus group served as a pilot (NI n = 5, ROI n = 4), but, due to its wealth of data, contributed to the final sample. Forty organisations were contacted in total by email with follow-up phone calls (if a phone number was available). Of the non-responders (n = 19), all had been contacted by email/through a contact form on their website only. A further nine organisations responded to initial communication and expressed being unable to help. Of those who could not help, six provided a reason. Reasons included: perception by the gatekeeper that parents would not be interested/willing, ongoing research involvement in other projects, unsuitable time of year and perception that participants not eligible. Seven organisations proceeded with one or more focus group(s) via their organisations, while one attempted to arrange one but received very little interest. Table 1 conveys the data collection broken down by recruitment strategy, excluding one pilot focus group which consisted of a convenience sample.

**Table 1:** Recruitment for the study based on each strategy employed

Recruitment strategy	Outcome
(i) Collaborator agreement contacts	Four organisations contacted, one focus group completed (n = 6, ROI)
(ii) Identifying additional relevant organisations	Seventy organisations contacted, fifteen focus groups completed (n = 65)
(iii) Social media	Two focus groups (n = 8)^a^

^a^Two participants recruited through social media joined a pre-arranged focus group that took place in a subsequently identified organisation

In NI, the team contacted 34 organisations by email with follow-up call where possible. Two organisations responded to initial emails but they could not help as their policy didn’t allow them to be involved in research. Five asked to be forwarded the study material in case they had any parents interested in taking part, but they did not respond subsequently. Twelve organisations sent positive replies and a visit by a member of the research team was arranged; eight of them proceeded with a focus group.

A breakdown of the sociodemographic characteristics of the participants who attended focus groups in ROI and NI can be seen in Table 2.

**Table 2:** Participant characteristics

Parent characteristic	N (ROI)	% (ROI)	N (NI)	% (NI)
Place of birth				
Ireland/Northern Ireland	33	71.7	34	91.9
Other part of United Kingdom	1	2.2	1	2.7
Other European country	4	8.7	1	2.7
Africa	6	13	0	0
Asia	1	2.2	0	0
Australia	0	0	1	2.7
Not specified	1	2.2	0	0
Ethnicity				
White	34	73.9	36	97.3
Irish Traveller^a^	5	10.8	0	0
Black or Black Irish: African	5	10.8	1	2.7
Any other Black background	1	2.2	0	0
Asian	1	2.2	0	0
Employment				
Employed / self-employed	18	39.1	26	70.3
Unemployed / home duties	25	54.3	10	27
Long term sickness/disability^b^	1	2.2	0	0
Student^2^	2	4.3	0	0
Not specified	0	0	1	2.7
Marriage status				
Married / cohabiting	36	78.2	24	64.9
Single	4	8.7	10	27
Separated / divorced^b^	0	0	3	8.1
Not specified	6	13	0	0
Eligibility for benefits				
Full medical card^b^or Healthy Start^c^	24	52.1	16	43.2
Social support				
Enough help	29	63	30	81.1
Not enough help	8	17.4	6	16.2
No help at all	3	6.5	1	2.7

^a^An indigenous ethnic minority group

^b^Asked in ROI only; Full medical card: a means tested entitlement to reduced cost or free medical care for a wide range of services used as an indicator for low income

^c^Asked in NI only; Healthy Start: a means tested UK government food welfare scheme for low income or at risk families used as an indicator for low income

Some questionnaire items differed slightly in each jurisdiction as they were taken from either the UK or Irish censuses (Additional file 2)

Additional file 3: Table S2 provides an overview of the number of indicators of disadvantage met by participants.

Most communication with parents was via the gatekeeper and, when it involved many steps, it was difficult to maintain contact. Moreover, when describing the process to gatekeepers, the additional stage of screening questions for participants (i.e. sending the questionnaire to participants, follow up, determining eligibility and communicating same to parents), may have been too burdensome, perhaps explaining the number who did not get back in contact. Importantly, the possibility of excluding some parents within their group or programme did not appeal to some organisations. Thus, we decided to recruit via invitation to pre-arranged focus group (as opposed to scoping out potential participants for screening and then making arrangements). This meant asking parents to self-screen and allowed for a reduced burden for gatekeepers, enabling parents decide whether to attend or not. While this process risked attendance by parents outside of the target population, it was more practical and time efficient for both the gatekeepers and the research team.

In NI, the recruitment approach was adapted to avoid overlap with another study with the same target population, to avoid research fatigue, which facilitated recruitment. Some challenges included services finishing up for summer, and in one case, a query around governance. Taking note of parents’ logistical preferences wherever possible allowed the research team to work around the majority and maximise attendance.

We found that giving as much information as concisely as possible about the study was key when contacting community organisations (gatekeepers) for help with recruitment. Speaking by phone rather than email also proved more productive (seen in the high number of non-responders being those contacted by email only), and allowed researchers to build rapport more easily.

Buy-in and interest from gatekeepers were deciding factors in whether or not information about the study would reach parents. It was sometimes clear that gatekeepers felt it important to protect their potentially vulnerable group from what they may have perceived as a risk of feeling inadequate or the perception of being accused of doing something 'wrong'. Programmes more familiar with participating in research were usually more willing to engage. Some gatekeepers sought resources from the research team in the form of information sessions or workshops around infant feeding or child nutrition in return for informing parents about the study, which helped with forging a relationship and a sense of reciprocal input. Arranging groups to coincide with events (such as workshops) already planned for parents helped attendance. Table 3 summarises the barriers to recruitment in this population that we encountered and describes solutions we found and suggests potential solutions for future research.

**Table 3:** Summary of recruitment challenges we encountered and suggested solutions

Recruitment challenge	Potential solution
Recruiting insufficient numbers in locality of research institutions and therefore needing to recruit nationwide	Ensure considerations and resources for travel or alternatively online data collection are built into grants applications, research protocols and timelines [17] Consider inclusion of a Public and Patient Involvement (PPI) panel to advise all aspects of the study, including recruitment [18]
Difficulty adhering to research timelines due to unsuccessful early recruitment efforts	Ensuring flexibility in terms of time and contingency plans, and Allowing for transcription and data analysis to run concurrently with further recruitment and data collection [19]
Challenges with capturing the attention and interest of gatekeepers during initial contact and building rapport	Contact by phone and not email Utilising existing connections where possible Providing study information as succinctly as possible Consider offering resources, data collection relevant to the goals of the organisation, or expertise [20] (in our case infant feeding workshops/information) to allow a sense of reciprocal input
Maintaining buy-in from gatekeepers due to the complicated logistics of carrying out screening questionnaires prior to inviting eligible participants to a focus group, and the possibility of excluding people	Avoiding too many steps in the process and pre-empting logistical barriers [20]: consider providing the inclusion criteria with details of pre-arranged focus groups, allowing participants to self-screen. Demographic questionnaires during data collection can be used to measure eligibility
Difficulty recruiting via social media compared with other routes: difficult to find appropriate groups/pages to target, with correct demographic and sufficient reach	Consider whether sponsored advertisements on social media may be helpful, and build this into research budget Approaching group/page administrators to post material the group to engender sense of legitimacy and relevance [21] Identifying social media ‘champions’ who could assist in online dissemination [22]
In areas with limited organisations to contact for recruitment (such as NI), there was a risk of research fatigue among those who are regularly approached for research	Consider whether collaboration with another research study to combine data collection for answering multiple research questions is feasible, while carefully assessing burden on the participant
Seasonality of services and participant availability e.g. parent group closing for the summer	Make a list of organisations and their schedules early on so approaching those who are seasonal can be prioritised [23]
Approaches used successfully recruited female parents/carers but did not result in recruitment of fathers/male carers	Additional and specific recruitment efforts for recruiting male parents should be researched and planned in advance, where fathers/male carers are explicitly invited [24]

### Discussion

Our findings demonstrate that using as wide a variety of communication tools was central to successful recruitment, and often direct communication by phone was best for reaching busy community groups. Social networking sites, whilst widely utilised among young people and a well-established recruitment avenue [25], were not a straightforward outlet for recruitment to health promotion research, particularly for capturing a specific target population such as ours. If research budgets allow, it may be worthwhile to pay for targeted advertising on social media, which has been shown as a useful research recruitment tool [26]. Some literature suggests that social media use is not representative of the general population [27] and this further emphasises the need for targeted approaches for recruitment online.

Recruiting participants from deprived populations through community organisations is recommended in the literature [14], and was efficient for accessing large groups of parents. It also added a sense of legitimacy to the study when being pitched to potential participants through someone with whom they were familiar. However, recruiting through such initiatives risks over-burdening gatekeepers and potentially service-users, and in research fatigue [28]. This may help explain, at least partly, the poor response rate to initial contact. Another consideration is that such organisations tend to exist in densely populated areas, and this is reflected in our low number of focus groups in rural areas compared with urban areas. Parents experiencing marginalisation who live rurally may be further isolated by living further from amenities.

## Limitations

We almost certainly encountered missed opportunities to include the voices of those who might not have the capacity or desire to attend community initiatives. Future research should explore additional strategies to include those from marginalised populations who are not in touch with local organisations, whilst being mindful of ethical considerations. Further, almost a third of participants did not meet eligibility criteria, resulting from our amendment to have potential participants self-screen and asking gatekeepers to invite participants to a pre-arranged focus group. This reduced our ability to manage how eligibility criteria were communicated to parents.

No participants availed of travel cost reimbursement. It is possible that some may not have had the means to pay for transport up front, or that asking for reimbursement risked feelings of shame or stigma. A consideration for future studies may be to offer to arrange transport in advance.

For the most part, we used proxy measures for indicators of disadvantage such as eligibility for income support, however this was in order to reduce the burden on participants. Among the research team, there was consensus that developing a questionnaire item to assess income was potentially intrusive and unnecessary, when eligibility for a medical card (in ROI)/Healthy Start (NI) was a good indicator. For some of the other indicators however, the relationship was less clear. We asked people to report their ethnicity and country of birth using items from the Irish census, however these alone do not give a clear picture of disadvantage.

With the exception of one male participant, this study did not capture father-driven data. This is an important factor to consider for future research, as family dynamics are extremely complex and all perspectives are important [29]. The help-seeking behaviours of male caregivers may differ, in that the services utilised by mothers/female caregivers may not be accessible or attractive to fathers for a variety of reasons [30].

In summary, key facilitators for recruitment were building rapport with gatekeepers, simplifying the recruitment process to reduce the burden for gatekeepers, and being flexible by amending the recruitment strategy in response to barriers encountered throughout the process. Specific recruitment strategies aimed at fathers and rural parents should be considered.

## Supplementary Information

**Additional file 1: Table S1.** Participant eligibility criteria.

**Additional file 2:** Participant demographic questionnaire.

**Additional file 3: Table S2.**Number of criteria associated with disadvantage met among participants.

## Data Availability

Not applicable.

## Abbreviations

ROI: Republic of Ireland
NI: Northern Ireland
