# Supplementary material for: Recruiting ‘hard to reach’ parents for health promotion research: experiences from a qualitative study
Source: BMC Res Notes. 2021 Jul 21;14:276. doi: 10.1186/s13104-021-05653-1 (PMC8293495; doi:10.1186/s13104-021-05653-1)
Supplement: Supplementary file 1 — Additional file 1: Table S1. Participant eligibility criteria. [file 13104_2021_5653_MOESM1_ESM.docx]

Table S1 Participant eligibility criteria

| (i) Having a baby aged 3-14 months and (ii) having one or more of the following: |
| --- |
| - a medical card (a means-tested entitlement to exemption for certain healthcare costs in the Republic of Ireland (ROI)) |
| - a disability |
| - belonging to a single-parent family |
| - being unemployed |
| - residing in social housing/direct provision (accommodation in ROI provided by the state to people seeking asylum) |
| - ROI: receiving an allowance from the State (except child benefit for which all parents are entitled) - Northern Ireland (NI): qualifying for Healthy Start (a government food welfare scheme for young parents and low-income families) |
| - being a member of an ethnic minority group |
| - being aged 20 years or younger |
| - non completion of secondary education |
| - low self-reported social support. |
