# Supplementary material for: Recruiting ‘hard to reach’ parents for health promotion research: experiences from a qualitative study
Source: BMC Res Notes. 2021 Jul 21;14:276. doi: 10.1186/s13104-021-05653-1 (PMC8293495; doi:10.1186/s13104-021-05653-1)
Supplement: Supplementary file 2 — Additional file 2: Participant demographic questionnaire. [file 13104_2021_5653_MOESM2_ESM.docx]

Weaning on the island of Ireland

Questionnaire

The following questionnaire will help us to understand a little bit more about you, your baby and your family, which will help our research. There are also some questions about healthy eating at the end. All of your answers will be kept **completely** **anonymous** as your name will not be written anywhere on it.

Please fill out the following questionnaire as best you can. If you are unclear of anything, please ask the researcher to help you. If you would prefer, the researcher can fill out the questionnaire with you.

**You do not have to answer ANY question you don’t want to. Please feel free to leave a question blank if you wish.**

THANK YOU.

| 1. Baby’s age |  |
| --- | --- |
| 2. Baby’s gender | □ Boy □ Girl |
| 3. Birth weight | *______ lbs ______ ounces* ***OR***  *______ kgs* |
| 4. In general, how would you describe the baby’s health **at birth?** | □ Very healthy, no problems  □ Healthy, but a few minor problems  □ Sometimes quite ill  □ Almost always unwell |
| 5. In general, how would you describe the baby’s health **now?** | □ Very healthy, no problems  □ Healthy, but a few minor problems  □ Sometimes quite ill  □ Almost always unwell |
| 6. Can you tell me whether your baby has received…  (please tick each one they have received) | □ Their six-week check-up  □ Vaccinations at 2 months  □ Vaccinations at 4 months  □ Vaccinations at 6 months  □ No vaccinations |
| 7. Was your baby ever breastfed (including colostrum)? | □ No →  *(go to next question)*  □ Yes  ↓  How long was (s)he breastfed for?  ____________*(days)* ***OR***  *____________(weeks)* ***OR***  ____________*(months)* ***OR***  □ Still breastfed  ↓  How long was (s)he **exclusively** breastfed for? *(this means no other food or drinks at all)*  ____________*(days)* ***OR***  *____________(weeks)* ***OR***  ____________*(months)* ***OR***  □ Still exclusively breastfed |

| 8. What age was your baby when you started weaning (introducing solid foods)? | *_________________ (age in months)*  □ I have not yet started |
| --- | --- |
| 9. What were the first solid foods your baby tried? | *_______________________________________* |
| 10. Are you the legal parent / guardian, who usually provides care for the baby? | □ No □ Yes |
| 11. Was the baby born early, late or on time? | □ Very early (32 weeks or less)  □ Somewhat early (33-36 weeks)  □ On time (37-42 weeks)  □ Late birth (43 weeks or more) |
| 12. Has a medical professional ever told you that your baby has any digestive allergies (eg. lactose intolerance)? | □ No □ Yes *(please give details)* |
| 13. Has your baby ever been diagnosed with any medical condition which affects his/her ability to feed, swallow or digest foods? | □ No □ Yes *(please give details)* |

**You, your family and your home**

| 1. Your gender? | | | Male □ Female □ | | | | | | | |
| --- | --- | --- | --- | --- | --- | --- | --- | --- | --- | --- |
| 2. **Your** date of birth? | | |  | | | | | | | |
| 3. Which of these best describes relationship to the baby? | | | □ Biological mother / father  □ Adoptive mother / father  □ Foster mother / father  □ Step-mother / step-father / partner of parent  □ Aunt / uncle  □ Grand parent  □ Other relative / in law  □ Unrelated guardian | | | | | | | |
| 4. In what country were you born? | | | □ Ireland (Republic)  □ Northern Ireland  □ Other UK  □ Other ______________________ | | | | | | | |
| 5. If you weren’t born in Ireland, in what year did you move to Ireland? | | | ____________ *(year you moved to Ireland)* | | | | | | | |
| 6. In comparison with an average person, would you say your health is…? | | | □ Excellent  □ Very good  □ Good  □ Fair  □ Poor | | | | | | | |
| 7. How would you rate your quality of life? | | | □ Very good  □ Good  □ Neither good nor poor  □ Poor  □ Very poor | | | | | | | |
| 8. What is your ethnic or cultural background? | | | | □ White: Irish  □ Irish Traveller  □ Any other White background  □ Black or Black Irish: African  □ Any other Black background  □ Asian or Asian Irish: Chinese  □ Any other Asian background  □ Other including mixed background  *(insert your own description)* | | | | | | |
| 9. What is the highest level of education you have completed to date? | | | | □ None / primary not complete  □ Primary or equivalent  □ Intermediate / junior / group certificate or equivalent  □ Leaving certificate or equivalent  □ Diploma / certificate  □ Degree or higher | | | | | | |
| 10. Do you qualify for a medical card?  *(If one of your children does but you don’t, please tick ‘no’)* | | | | □ Yes, full medical card    □ Yes, GP only medical card    □ No | | | | | | |
| 11. What is your current marital status? | | | | □ Single (never married)  □ Cohabiting (living with partner)  □ Married  □ Separated  □ Divorced  □ Widowed | | | | | | |
| 12. Which of these descriptions BEST describes your usual situation in regard to work? | | | | | | | | | | |
| □ Employee (including apprenticeship or community employment)  □ Self-employed outside farming  □ Farmer  □ Student full-time  □ On state training scheme | | | | | | □ Unemployed, looking for a job  □ Long-term sickness or disability  □ Home duties / looking after the home or family  □ Retired  □ Other___*____________________* | | | | |
| 13. How many children do you have? | | 1 □ 2 □ 3□ 4 or more□ | | | | | | | | |
| 14. How many people live in your home? | |  | | | | | | | | |
| 15. Is your home…? | | □ Rented from local authority  □ Rented privately  □ Owned outright  □ Owned with a mortgage | | | | | | | | |
| 16. Which of the following best describes where you live? | | □ Countryside  □ Village  □ Town  □ City  □ Other _________________________ | | | | | | | | |
| 17. Overall, how do you feel about the amount of support or help you get with your child(ren), from family or friends living outside your household? | | □ I get enough help  □ I don’t get enough help  □ I don’t get any help at all  □ I don’t need any help | | | | | | | | |
| 18. Are you in regular contact with the baby’s grandparents? | | □ No  □ All grandparents are deceased  □ All grandparents live abroad  □ Yes *(please answer next question)*  *↓* | | | | | | | | |
| 19. **If you said yes for question 18**, here are some questions about how much support you receive from your baby’s grandparents. Please tick the box that best indicates how often the baby’s grandparents do each activity mentioned along the side. | | | | | | | | | | |
| **How often do the baby’s grandparents:** | **Never** | | | | **Less than once every 3 months** | | **At least once every 3 months** | **At least once a month** | **At least once a week** | **Every day or almost every day** |
| Babysit? |  | | | |  | |  |  |  |  |
| Stay overnight? |  | | | |  | |  |  |  |  |
| Take the baby out? |  | | | |  | |  |  |  |  |
| Buy clothes or toys for the baby? |  | | | |  | |  |  |  |  |
| Help you around the house? |  | | | |  | |  |  |  |  |
| Help you out financially? |  | | | |  | |  |  |  |  |
