# Supplementary material for: Recruiting ‘hard to reach’ parents for health promotion research: experiences from a qualitative study
Source: BMC Res Notes. 2021 Jul 21;14:276. doi: 10.1186/s13104-021-05653-1 (PMC8293495; doi:10.1186/s13104-021-05653-1)
Supplement: Supplementary file 3 — Additional file 3: Table S2.Number of criteria associated with disadvantage met among participants. [file 13104_2021_5653_MOESM3_ESM.docx]

Table S2 Number of criteria associated with disadvantage met among participants

| Number of eligibility criteria met by participants | ROI  % (n) | NI  % (N) | Total  % (n) |
| --- | --- | --- | --- |
| 0 | 22 (10) | 35 (13) | 28 (23) |
| 1 | 18 (9) | 22 (8) | 20 (17) |
| 2 | 7 (3) | 14 (5) | 10 (8) |
| 3 | 26 (12) | 19 (7) | 23 (19) |
| 4 | 17 (8) | 5 (2) | 12 (10) |
| >4 | 9 (4) | 5 (2) | 7 (6) |
